# Young adults’ understanding of, and reaction to, those with uncertain genetic results

**DOI:** 10.1007/s12687-026-00957-w

**Published:** 2026-10-20

**Authors:** Fiona Ulph, Kate Fifield, Grace Christie, Eleanor Horowitz

**Affiliations:** Manchester Centre for Health Psychology, Division of Psychology & Mental Health, School of Health Sciences, Faculty of Biology, Medicine and Health, Manchester Academic Health Science Centre, University of Manchester, Oxford Road, Manchester, M13 9PL UK

**Keywords:** Newborn screening, Uncertain results, Young adults, Peer, Qualitative methods

## Abstract

**Background:**

Genetic advances increase the likelihood that children may receive uncertain diagnoses such as cystic fibrosis screen positive inconclusive diagnosis (CFSPID) from newborn screening. Research suggests a profound impact on parents. Research is needed to understand the impact on the child, especially at key developmental points such as young adulthood, when identity forms. As young adults (YA) are influenced by peer reactions, this novel study explored YAs’ sensemaking of CFSPID, and their hypothetical reactions to peers or romantic partners with CFSPID.

**Methods:**

Four focus groups were conducted and analysed using thematic analysis across three sections: understanding, peer and romantic relationships.

**Results:**

Understanding: Without knowledge about CFSPID, participants used cystic fibrosis, genetic and contagious illness knowledge to form assumptions. Peer and romantic relationships: In both sections, YAs wanted to support their friend/partner; however, their responses revealed assumptions that a person with CFSPID would need more physical and psychological support and would be seen differently by some. Most wanted to learn more but were unsure whether it was appropriate to ask the person with CFPSID. Although rare, some reported they would avoid romantic relationships with YAs with CFSPID. All data suggesting people with CFPSID would be treated differently was based on erroneous assumptions due to a lack of CFSPID knowledge. Providing information about how CFPSID is different from CF did not appear sufficient to stop a reliance on CF knowledge when making sense of CFSPID.

**Conclusion:**

Further research should explore whether it’s possible to change assumptions when YAs disclose uncertain results.

**Supplementary Information:**

The online version contains supplementary material available at https://doi.org/10.1007/s12687-026-00957-w.

## Introduction

In screening, careful consideration is given to which conditions to screen for and balancing the benefits of timely diagnosis and treatment against the potential harms of screening, which can include triggering anxiety in parents (Ulph and Bennett 2022), over medicalisation (Sinha and Southern 2021), stigma (Ulph et al. 2011), and impact on identity (Lewis et al. 2010). When considering the return of carrier results or results of uncertain significance from newborn screening, the contested balance for diagnoses becomes a more nuanced debate. Research is needed to understand the impact of returning such results (Ulph and Bennett 2022). Furthermore, it has been argued that when considering the health impact of newborn screening, we should examine both physical and psychological impacts (Ulph 2023), and that it is important to examine the impact not only on parents but also on the child in the longer term (Bowen et al. 2024).

### Stigma towards those with positive NBS results

Link and Phelan’s (2001) framework of stigma stages including labelling, negative stereotypes and separation would be key behaviours of concern if seen in young adults when trying to establish peer and romantic relationships. Those who receive a genetic diagnosis can be concerned that they will be stigmatised (Etchegary 2007) and stigma has been shown to be experienced by those with hereditary conditions (Oliveira et al. 2025). Although no research has examined whether people receiving uncertain NBS results experience stigma, research has shown that those receiving carrier results may experience stigmatisation and discrimination (Baynam et al. 2024; Bowen et al. 2025). Furthermore, research has shown that carriers experience both self (Stamatoyannopoulos 1974) and social stigma (Evers-Kiebooms et al. 1994; Lewis et al. 2010). Social stigma occurs when differences are labelled as undesirable, resulting in discrimination (Bathje and Marston 2014), whilst self-stigma occurs when the individual internalises the stereotypes made about their group (Corrigan et al. 2010). As Bowen et al. highlight, all of these studies were conducted with adults who received genetic counselling. However, when trying to understand the impact on the child identified via NBS, they and their peers, who will determine to an extent the level of stigma experienced, are not likely to receive information from specialist health professionals. Studies which have looked at peer reactions to carriers have found that children (aged 10–17) views were stigmatising, and carriers were seen as ‘different’, with beliefs that they would have lowered self-concept and would not fit into their peer group (Noke 2015). These results were echoed in a study with young, scientifically literate adults, which found that they held stigmatising beliefs about carriers even after spending time reading NHS information explaining carrier status before their interviews (Bowen et al. 2025) suggesting that information provision to individuals alone is not sufficient to change stigmatising beliefs in novel situations. Furthermore, they not only expressed their own negative views but also believed that others would hold negative views of carriers and were aware of others’ judgments. Thus, although carrier status results are considered medically benign, they may not be psychosocially benign (Ulph and Bennett 2022).

### The impact of uncertain results in newborn screening

Although screening for cystic fibrosis via NBS has led to positive outcomes for those with diagnoses, it has raised questions about how to manage scenarios where there is an uncertain result (Barben and Southern 2016). The US named these CF transmembrane conductance regulator (CFTR)-related metabolic syndrome (CRMS), whilst European countries used Cystic Fibrosis Screen Positive Inconclusive Diagnosis (CFSPID). These were harmonised so that CRMS/CFSPID is now used(Barben et al. 2021). The US has been returning these results since 2009, meaning children would be 17, whilst in Europe, the term was used from 2015, and so children would be 11. There is a lack of clarity over the number of people receiving this designation, as countries employ different follow-up testing protocols and often adapt them to reduce the identification of CFSPID.

The return of CFSPID designations following newborn screening has previously been used as a case study to examine the impact of uncertain results on parents (Johnson et al. 2022), however, the CFSPID literature that is available purely focuses on the impact CFSPID will have on the parents (Loukou et al. 2024). This shows us that parents of children in the CFSPID group raised concerns about the future emotional and behavioural development of their children (Perobelli et al. 2009). Furthermore, research suggests that confusing communication about CFSPID led to parents concentrating on the ‘CF’ part of CFSPID rather than “inconclusive diagnosis”, and even when parents did understand the uncertainty, they found themselves reverting back to using CF terminology such as ‘atypical’, ‘mild CF’, or ‘on the border’ (Tluczek et al. 2019 p. 302). This could be triggered because so few people had heard of CFSPID (Johnson et al. 2019), but it raises concerns that, in turn, children will internalise an identity of having a condition. A similar pattern of findings is emerging from the nascent field of parents’ experiences of receiving uncertain results via SCID screening (Raspa et al. 2023) who respond to the diagnoses in a similar manner to those receiving a positive SCID result.

### How do young adults make sense of uncertain/unclear diagnoses?

Although little is known about parents’ plans to explain uncertain results to their children, the teenage years are a key time when parents pass on genetic information to their children (Ulph et al. 2014; Forrest Keenan et al. 2015; Goldman et al. 2018; Pieper et al. 2020). Furthermore, when considering the impact of uncertain NBS diagnoses on the children receiving them, emerging adulthood (a pivotal life stage; 18–25 years old; (Arnett 2000) is a critical time for identity formation as individuals explore love, work, and world views. Specifically, peer and romantic relationships are central to the formation of young adult identity (Joshi and Deuskar 2016). Unfortunately, recent work has shown that those with conditions report impacts on their romantic and peer relationships (Oliveira et al. 2025). Thus, this study sought to examine young adults’ views towards a peer who has been given a CFSPID designation.

## Method

### Study design

FGs were used, as not only do they enable an exploration of people’s attitudes by mimicking the natural processes of communication, where participants listen, discuss, and can develop ideas collectively, but they also enable researchers to observe the social factors that influence opinions (Wilkinson 2004).

FGs can facilitate discussion, especially in young adults, as they feel supported by their peers, which balances the power between themselves and the researcher (Farquhar and Das 1999). The materials used were developed by the study team, which included an experienced CFPSID researcher, following a review of the literature. These were not piloted.

#### Demographic questionnaire

This gathered information on participants’ age, gender, year of study, A-level results, whether they had children and their previous experiences with CFSPID, CF and genetics education. These forms were placed on the table before the focus group and linked to participants only via a study identifier. The participants completed these and handed them back to the researcher.

#### FG schedule

The FG schedule explored participants’ preconceptions about both the CRMS and CFSPID acronyms and their initial views on the characteristics of individuals with CFSPID. It then explored the participants’ reactions to hypothetical disclosure from peers or a romantic partner and their related information-seeking behaviours.

#### Topic information sheet

Two PowerPoint slides with information on CF, cited from the NHS England website and information on CFSPID, cited from relevant literature. Ethical approval was granted for this study by the University of Manchester Ethical Review Board (ref: 2020-8060-12862).

### Participants

40 participants aged 18–25 years were recruited via convenience sampling through their university course credit advertising system. Using a pre-FG questionnaire, participants were excluded if they had prior experience of, or knew someone with CF, which was a stipulation of the ethics committee, and to ensure that participants represented the typical peer who is unlikely to have much awareness of CFSPID. All participants received the Participant Information Sheet by email 24 h before the FG.

### Procedure

Before the FG, the participants read a study information sheet, could ask questions and signed consent forms. All FGs were held on university premises and were attended by a facilitator and one assistant. The facilitator started the group with a brief introduction about what would be involved in the session, then introduced the topics. The facilitator guided the FG topic discussion using the pre-designed schedule, including prompts and encouraged open discussion. The facilitator also engaged with less-vocal participants to encourage them to participate, whilst the assistant took notes during the group and monitored participants for any signs of distress. During the session, participants were asked to complete exercises to facilitate discussion by writing down their responses to questions and then sharing them with the group. After the preconception section of the FG, participants were shown information slides on CF and CFSPID and further discussion was held on their views and reactions. All FGs were audio recorded. Where erroneous beliefs were raised in the focus groups, these were not corrected during the group, but were at the end. All participants received a debrief sheet as standard, which signposted routes for support/further information.

### Data analysis

Each FG was transcribed verbatim and anonymised. Reflexive thematic analysis was used (Braun and Clarke 2021) as it has been shown to be appropriate for health research and FGs (Braun and Clarke 2014). An inductive semantic approach was followed, in which codes were developed straight from the data set with meaning prioritised. Themes were identified with a realist stance, in which one assumed that participants could provide direct access to their true experiences and meanings through the language they used (Clarke et al. 2015). The transcripts were managed in Microsoft Word, and the first three were coded by each researcher, with the fourth being shared between them. As this was an undergraduate student project, each researcher took one section (understanding, peer relationships and romantic relationships) to write up, but as they were coding entire transcripts, they coded across all areas. The researchers then met with the project supervisor to reach consensus on each section’s codes and discuss emerging themes. The final stage was for each researcher to meet separately with the supervisor to finalise codes and themes for their own sections. There were a few references in each FG that appeared to be influenced by the participant information sheet, were coded as such, and removed from analysis. This was because this study was interested in people’s reactions to and understanding of an unknown diagnosis, who, in reality, would not receive pre-disclosure information. Reflexivity was practised throughout. The researchers believed that uncertain diagnoses could provide health benefits, but they were aware of peers’ reactions to carriers and were curious to see how peers would respond to uncertain diagnoses.

## Findings

A total of 36 participants were recruited to take part in four FGs lasting 23–57 min. The first FG had technical issues, so only the first 23 min were recorded. Each FG included 9 participants aged 18–24 years, of mixed gender. The participant characteristics are summarised in Table 1.

**Table 1:** Characteristics of Focus Group Participants (n=36)

Characteristics of Focus Group Participants (*n* = 36)		
Characteristic	Number of Participants	
*Gender*		
Female	33	
Male	3	
*Age*		
18	13	
19	17	
20	3	
21+	3	
*Year of Study*		
Year 1	30	
Year 2	6	

 Because the analysis was split by topic, the results are presented in 3 sections: understanding, peer relationships and romantic relationships. However, due to the interrelated nature of the topics, we sometimes refer to other sections to avoid repetition of data and narrative. Themes and their subthemes are presented in Table 2.

**Table 2:** Note: This is mandatory. Please provide

Section	*Preconceptions and Information Seeking*		*Peer Relationships*		*Romantic Relationships*		
Themes	Uncertainty Sensemaking	Seeking Understanding from External Information	Existing Friendships Models	Inner Freakout	Disclosure in the Relationship	Divergence from Relationship Norms	Impact of Knowledge on Relationship
**Subthemes**	*Searching for clues*	*Difficulty in disclosing*	*Desire to support*	*Psychologically challenging*	*Worries for the one disclosing*	*Concerns about supporting partner*	*Changes in their attitudes towards partner with CFSPID*
	*Symptom-driven perception*	*Personal sources of understanding*	*Burden with questions*	*Disclosure changes friend’s behaviour*	*Consideration for partner*	*Uncertain future*	*Reactions of people external to relationship*
	*Existing models driving understanding of CFSPID*	*Textbook sources of understanding*		*Stigma unintentionally happens*			*Relationship avoidance*

### Understanding

#### Uncertainty sensemaking: Searching for clues

None of the participants had heard of CFSPID. This led participants to search for clues in the acronym. In some participants, the very use of an acronym triggered a possible connection to genetics, as they were aware of genes being shortened and using acronyms to describe conditions *“Obviously*,* the letters it kind of well seemed like genetic disorder”* (P8, FG1). Recognising that genetic labels are often acronyms, participants began to assume the nature and severity of the condition *“well*,* I thought it would be lifelong because you can’t get rid of your genes if you have them”* (P3, FG4). The participants also searched for clues within the acronyms themselves. ‘CF’ was recognised by some participants.

*“It’s just like CF is not from what I’ve like heard of and studied*,* there’s not many genetic disorders with them two letters that are identifiable by that disease*,* if that makes sense?”* (P7, FG1).

This participant reported having studied CF in school. which is perhaps the reason why she recognised the letters. One participant also agreed they had learnt it in school, but ‘not in detail’ (P2, FG1), whilst others did not mention they had learnt about it in school. Along with CF being recognised, within the CRMS acronym, ‘MS’ was recognised by a few participants as standing for Multiple Sclerosis. This led to participants in FG4 predicting MS symptoms for CRMS.

#### Uncertainty sensemaking: symptom-driven perception

After receiving information about CFSPID, one participant in FG3 (P5) noted that CFSPID patients were not always symptomatic and may not develop CF symptoms. This led other participants in FG3 to report that this may have a positive impact on the individual, including no changes to friendships, relationships, social aspects, and their own well-being.

*“And then if they don’t have any symptoms*,* I don’t think it would change how you would act when you were together and the friendship you have.”* (P1, FG3).

Thus, lack of symptoms was equated with a more positive life experience. A few participants built on this symptom-driven perception by reporting that, in the absence of symptoms, individuals with CFSPID would want to be, or be seen as, normal. *“I think depends on the personality as well*,* some people would just hide and just act normal.”* (P2, FG3). However, this suggests that underlying these statements is the belief that individuals with CFSPID are not normal, because you would not have to ‘act’ or “hide” to be considered normal, and through this they are placing them in a different group (the precursor of discrimination). The majority of participants, however, ignored that individuals with CFSPID would not always develop symptoms (despite being shown materials that stated this during the group) and assumed they would be symptomatic constantly, which would negatively impact on the individual’s life, whether mentally, physically, or socially.

*“Because you’re kind of like you’ve been given some diagnosis that is potentially disabling*,* so you’ve kind of got limiting potential that’s been giving to you unfairly*,* so you feel like maybe you have to work harder*,* so you’re less confident that you’re able to achieve as much as others who don’t have that genetic disorder”* (P7, FG1).

This piece of data typifies responses that started by recognising CFSPID as only potentially a condition, but morphs into talking about having a condition, suggesting a sway towards a binary model of illness where one is ill or well. It also shows again that although not specifically mentioning stigma, they are referencing people as CFSPID as having characteristics that are undesirable (less confidence).

#### Uncertainty sensemaking: existing models drive understanding of CFSPID

Once the link to CF was made, either by recalling the pre-study information sheet or by clue-searching in the acronym, the participants used their existing knowledge of CF to try to establish what CFSPID is.

*“[M: how serious do you think CFSPID might be or not?] I think pretty serious just from what I’ve heard about cystic fibrosis before”* (P5, FG4).

As they understood little beyond the ‘CF’ part of CFSPID, the participants relied on their CF knowledge, with many avoiding discussing the ‘SPID’ part. Participants then applied their knowledge of CF symptoms to individuals with CFSPID and again differentiated from “normal” people:

*“In theory*,* yeah*,* but also I feel like um there’s quite a lot they might physically struggle with*,* like breathing difficulties and I feel like you can’t exert yourself as much as a normal person something like that maybe”* (P2, FG1).

The majority of participants focused on respiratory issues associated with CF and how this could impact an individual’s life. Other common narratives focused on loneliness and feeling different, but all were discussed as negative limitations on an individual’s life. Even after information about CFSPID was provided, participants still tended to focus on a negative view, focusing on CF symptoms rather than the individuals with CFSPID being asymptomatic. This focus on CF symptoms was exaggerated in some participants who seemed, as the FG progressed, to forget that we were discussing CFSPID and not CF.

*“We kind of focused on the idea that cystic fibrosis*,* cystic fibrosis is based on an issue with the respiratory system. So*,* like like*,* physical*,* you might like*,* coughing*,* issues. Erm*,* but then I think like they have a machine at night probably. That can help them breathe”* (P5, FG3).

This led participants to discuss their opinions solely through a CF lens, rather than discussing the differences between CF and CFSPID. Whether this was because they did not want to appear to lack knowledge and so shifted the topic to something they did know (CF), or they were unaware that CFSPID is not CF even after being informed, was unclear. However, one participant did pick up on the fact that CFSPID is not CF and explained how, not knowing what CFSPID was, she could not assume anything about its nature: *“If these are like genetic*,* then surely*,* they are like different to normal cystic fibrosis”* (P7, FG3). According to the pre-study questionnaire, P7 (FG3) had read books on epigenetics. This may be why she picked up on the genetic differences between CF and CFSPID and therefore assumed (correctly) that CFSPID is different to CF.

Another narrative was the fear of contagion driven by a general misunderstanding of genetic conditions.

*“I think I’ve met a few people who are scared of catching it (CF) because they don’t quite understand the concept that it is a genetic disorder”* (P4, FG4).

*“Yeah*,* definitely*,* cos I think like when you tell people about a disease or something*,* the first question in the back of people’s mind is always like*,* is it contagious? Can I like develop this from you?”* (P7, FG2).

The participants expressed this fear in both relation to CF and general diseases, suggesting that their cognitive models about health diagnoses and CF are resulting in stigma against individuals with CFSPID. Again, although stigma is not mentioned directly, they are distinguishing people with CFSPID from others, including here, a sense that they may have the undesirable characteristic of being a threat to one’s health if contagious.

#### Seeking understanding from external information: difficulty in disclosing

The participants reported how they believed CFSPID would be difficult to explain and that this would impact how much others would understand about the condition.

*“Annoying to explain […] Um*,* well*,* I don’t know what is it to begin with*,* so like I assume possible a lot of people don’t know what it is so to explain to people what it is its must get like tiring to describe it to people*,* probably who can’t understand that much.”* (P3, FG4).

The participants also discussed how if the person’s health condition did not match the assumed symptoms, this could lead to issues with understanding *“You might feel like a bit confused because they seem fine*,* but then they’ll tell you that they’re not so…”* (P3, FG2). However, the participants were aware that discussing the condition might not be what the individual wants: *“I feel like I’d probably not wanna make it worse for them by throwing a load of questions at them”* (P7, FG4). This issue is expanded upon in the peer relationships section. However, participants warned that if people perceive that individuals with CFSPID do not want to talk about their condition, then that would lead to more difficult sensemaking using external sources and, therefore, may increase the inaccurate judgments discussed above. “*So*,* I guess it’s like if people aren’t informed and don’t know what it is*,* it can kind of could be scared of it.”* (P4, FG4). Highlighting a need for awareness of what designations mean, even if the person is currently not affected by them, to ensure people are not “scared” and do not treat them differently.

#### Seeking understanding from external information: personal sources of understanding

The majority of participants discussed approaching the individual with CFSPID for information to help them understand CFSPID, which is developed in the peer and romantic sections below, to explore the nuanced differences. Another personal information source that participants discussed was social media accounts:

*“Look for people that had it*,* like get a lot of people with social media accounts or blogs or stuff that talk about their illnesses*,* and they’re centred around breaking down stigma about those illnesses and educating people about them. So*,* I would probably look for something like that on the disorder.”* (P4, FG4).

Of interest, this was the only participant in FG4 that reported having current experience with friends who had complex chronic diseases (Cockayne syndrome and Coeliac disease). This participant was clear about what they were looking for and why they looked at personal social media accounts in this context. Another participant discussed how watching a film improved her understanding.

*“the girl*,* the main character in it*,* she like vlogs about her condition […] which I think is quite a good idea because before that I had an idea of what it was*,* but I think it was more the movie that told me exactly what it was*,* so in that sense it was good”* (P9, FG4).

This CF film was a topic throughout FG4, with the majority discussing its inaccuracies and unsuitability as an information source; ‘It’s a terrible movie’ (P5, FG4). But P9 reported that the social media aspect benefited her understanding of CF, and so suggested this type of information source could have similar benefits for CFSPID. As the FG schedule specified a friend and a romantic relationship disclosure, many participants assumed they could use the individual as an information source due to their relationship and ‘because they have some sort of trust in you’ (P3, FG3). However, one participant reported that using the individual with CFSPID as an information source depends on relationship quality: *“*If I was comfortable with the person who had told me, I would maybe ask them*”* (P10, FG1). This participant realised that not all disclosures occur between close friends, and so using the individual with CFSPID as an information source may not be suitable.

#### Seeking understanding from external information: textbook sources of understanding

Most participants discussed how they would seek further information about CFSPID in the scenario of a peer/romantic relationship disclosure. One way they believed they would do this was by seeking textbook sources “like a lot of you can find out yourself through research and stuff” (P7, FG4). Both websites and broader research were discussed, but firsthand accounts of CFSPID, such as those discussed above, were not included. This suggests that once a disclosure has been made, peers will want to seek out as much information as possible, but some participants were vague about where they would look and what they were looking for. Some participants spontaneously reported that they would look for textbook information themselves, as they did not believe their partner would tell them certain information.

*“And a lot of the time people don’t want to be a burden and say something’s wrong*,* so they could hold back and stuff as well”* (P10, FG2).

Participants believed that a partner with CFSPID might not disclose all the information about their condition and that they themselves would have to find out some of the facts. The main textbook cited was the NHS website, which was considered a more trustworthy source than others.

*“Like I suppose like when I look for medical advice online*,* NHS website um*,* like definitely that over Wikipedia say but that’s not to say I don’t use Wikipedia I definitely do [laugh] but um yeah I think if I really wanted to know like the symptoms and causes not causes its genetic but you know things like that NHS website”* (P2, FG1).

This implies that some participants were aware that not all textbook sources are equally accurate. But contrary to this, participants still reported they would use these perceived less-trusted textbook sources to gain information.

### Peer relationships

#### Existing friendships models: desire to support

Although participants were not explicitly asked a direct question about supporting their friend with CFSPID, this was a recurring topic they discussed naturally, where they expressed similar beliefs about their role as a friend and emphasised the importance of supporting their friend with CFSPID. Discussions about support were similar across the FGs, and participants unanimously agreed with statements about the necessity of supporting friends, indicated by both verbal comments and non-verbal cues, e.g. nodding.

*“I’d probably be psychological there [sic] so they don’t feel alone*,* cos obviously*,* if you’ve got a diagnosis like that the first thing you would be thinking is I don’t know how long I am going to live*,* or I don’t know what is happening or what is going to happen” (P1*,* FG3)*.

Again here participants can be seen to fall back on the language of “diagnosis” and concerns about prognoses, which might be relevant for some with CFSPID later, but will not be relevant for all. When referring to CFSPID, typically, participants reported pessimistic feelings, with two participants referring to the psychological element of support. Many believed that if their friend was diagnosed with CFSPID, this would have an adverse influence on life expectancy, and so supporting their friend was a key concern.

Although participants stated the difficulty in understanding or being able to relate to CFSPID, participants’ views were dependent on asking their friend with CFSPID how they could support them. This was evident in two of the FG discussions:

*“Like*,* how I can support them? Like what can I do*,* like ‘cause I would see them like quite often*,* if they were my friend*,* I would want to know how I could support them as much as I can.” (P5*,* FG4)*.

Participants’ responses appeared genuine, and respondents found it easy to discuss their beliefs about the desire to support and the position they would see themselves in.

#### Existing friendships models: burden with questions

There was some variation in responses amongst participants in relation to the consequences of questioning their friend with CFSPID about their diagnosis. In two FGs, participants were explicitly asked whether they had any questions they would ask their friend with CFSPID after the initial disclosure. Participants reported they would be reluctant to question their friend with CFSPID because of potential negative consequences.

*“I feel like I’d probably not wanna make it worse for them by throwing a load of questions at them*,* like a lot of you can find out yourself through research and stuff*,* and I think if it was me personally*,* I wouldn’t wanna bombard them with questions*,* ‘cause that would be seen as quite like an extreme reaction.” (P7*,* FG4)*.

Similarly, participants expressed that they would refrain from asking questions about life expectancy, again focusing on the idea that this is a scenario with a serious prognosis rather than an uncertain one: *“You wouldn’t want to say to them when are you going to die? Cos they don’t probably wanna know either” (P3*,* FG2).* However, in contrast, one participant believed it was better to ask their friend questions in order to fully understand:

*“I think I would [ask questions] just because I think like I would want to be completely honest with them and on the same page as well*,* and like everything*,* like they were finding out I’d want to find out*,* I’d want us to both know.” (P7*,* FG2)*.

Across all FGs, this was the only participant who stated the need to ask questions directly to the person with CFSPID. Other participants in this group did not express whether they agreed or disagreed with this viewpoint. This was a female participant who, on several occasions during the FG, stated opposing viewpoints to other participants and tended to elaborate on her opinions. In addition, she believed the desire for shared knowledge in a friendship was important, but it depended on how close you were with that friend.

#### Inner freakout: psychologically challenging

When participants were asked about how their friend’s diagnosis would make them feel, the majority of participants expressed that they would find it psychologically difficult. Participants identified several challenges due to the uncertainty of CFSPID. Difficulty in knowing how to act around and respond to uncertain diagnoses, versus knowing how to act if a diagnosis is given is highlighted below: :

*“[…] The uncertainty*,* then it would upset you. The fact that you don’t know or supposed* [sic] *to do in that situation. You don’t know how you’re supposed to act. Whether you’re supposed to act as if they do have it or don’t ask if anything has changed*,* ‘cause obviously there’s an uncertainty you wouldn’t know how to respond to it […]” (P1*,* FG3)*.

Similarly, due to the complexity of diagnostic uncertainty, participants in all FGs consistently reported that their reaction would be based on their knowledge that CF is life-limiting, and as such using diagnostic information in the face of uncertainty. *“Because I don’t really want my best friend to die and I’d be upset for her as well.” (P7*,* FG2).* Several participants stated they would likely experience distress and sadness, particularly because of their concerns about the impact of potentially losing a friend. Participants had previously been given factual information that those diagnosed with CFSPID are asymptomatic; nevertheless, participants tended to focus on the information that was relevant for those with a CF diagnosis instead. Thus, salient information such as CF and shortened life expectancy was frequently attended to when participants discussed their reactions to a CFSPID disclosure. What appeared to be happening was that in the absence of the ability to deal with uncertainty, participants were falling back on prior knowledge, even if this was more dramatic, distressing and not currently correct.

Furthermore, some participants had a negative initial inner reaction to a friend’s CFSPID disclosure:

*“Also*,* how they feel about it*,* ‘cause you don’t wanna like react*,* you wanna know how they are feeling before you freak out*,* I dunno.” (P7*,* FG4)*.

Participants described a sense of struggle combined with the complexity of not knowing how they were meant to react in this novel situation.

#### Inner freak out: disclosure changes a friend’s behaviour

Participants in all groups spontaneously suggested their behaviour would change following their friend’s CFSPID disclosure, without being prompted to reflect on this. Several participants suggested they would become more cautious about their friends’ needs as such seeing their friends as different.

*M: “Why would you be more worried about them compared if you were going for food with a friend without CFSPID?*

*P2: “Because yeah*,* because of digestive problems and stuff and if I would something which is quite*,* er exercise or something*,* you know. I probably wouldn’t be able to do that anymore with that friend.” (FG3)*.

There was no general agreement among other participants in this group, but when asked about other types of behaviours they would change, participants did comment on how they would take “more precautions” (P10, FG3) and be “overprotective” (P1, FG3) with their friend. Participants expressed the belief that they would change the way they do things with their friend.

*“Start to worry a lot about like the things they do*,* even unnecessarily*,* but like it’s probably not very helpful*,* but still.” (P2*,* FG2)*.

Participants recognised that treating their friend differently could have a negative impact on a friendship:

*“ If I had this or got this*,* I wouldn’t want people to treat me differently because of it*,* so I guess that would be something that they have to deal with*,* like people treating them differently about it*,* which would be like*,* yeah*,* not great.” (P9*,* FG4)*.

In contrast, across all FGs, one participant believed that a CFSPID disclosure would have no impact on their friendship or how they would behave.

*“Er yeah I have got a friend who’s got Crohn’s and er*,* I don’t really know about it but it’s kinda like similar in that it’s a disease*,* she*,* I let her tell me*,* I don’t act like act any differently*,* it’s kinda like she’ll let me know how she is feeling*,* but it doesn’t really affect how we are” (P7*,* FG3)*.

In the same FG, it is of interest that the only participant who suggested that her behaviour would not change after a friend’s CFSPID disclosure stated contradictory views elsewhere in the FG.

#### Inner freak out: stigma unintentionally happens

In all FGs, participants were asked about stigma, and they responded at length. Some participants believed that CFSPID was life-limiting and would interfere with an individual’s ability to do things.

*“I don’t know*,* if you’ve just told someone you have a disorder*,* a genetic disorder*,* I think the first thing brings to mind is a weakness in some area*,* but leads to being incapable in doing certain things*,* that’s all. That’s all that would come to my mind if I didn’t know anything.” (P2*,* FG3)*.

One commonality in all FGs was participants’ agreement about the impact of stigma. Participants stated that their lack of knowledge about CFPSID led to the belief that CFSPD was potentially life-limiting or terminal.

*“If you’re like uninformed about it*,* then you’d be like*,* well that person’s going to die*,* like if you don’t know very much about it.” (P2*,* FG2)*.

As discussed above, this lack of understanding could lead to fear for the person with CFSPID, and concerns that others might be wary of them. In contrast, two participants in different FGs had views that differed from most other participants. They instead believed that there is not much stigma attached to CFSPID because generally, people would not know or understand what it is or what it means.

*“I don’t know ‘cause like obviously if they say to you I have CFSPID*,* I’d just be like I have no idea what that is*,* so because it’s not that well known*,* there’s not much of a stigma around it […]”* (P7, FG2).

No data in the FG or contextual questionnaires could explain why these participants responded differently from other participants. Although differing views were apparent from participants, mostly participant views were centred around the belief that stigma was likely to be an issue for those diagnosed with CFSPID. Moreover, several participants emphasised the importance of education to prevent stigma.

### Romantic relationships

#### Disclosure in the relationship: worries for the one disclosing

Participants across all FGs held concerns about disclosing CFSPID to a partner. Participants reported that if they had CFSPID, they would be scared to tell their partner because of negative reactions suggesting that they believe this label is associated with negative stereotypes:

*“I can understand it’s something that would like be very difficult to tell someone*,* especially someone you want to plan a future because you’d be worried about whether they would stick iit out with you or like leave you for it*,* which obviously would be really hurtful and a painful experience”* (P9, FG4).

This participant implied that disclosing CFSPID would be serious enough to jeopardise a relationship, subverting the normal assumption that a serious partner would stay during difficult times, thereby situating CFSPID as a potential threat, not only to relationships, but also to a person’s emotional well-being. This participant expressed the common view that they would tell their partner, even though they believed it would be hard. A couple of participants voiced concerns about the moral dilemma they might be facing:

*“The conflict in their minds in terms of not knowing what they’re supposed to do and whether they should tell them or not […] at the same time they don’t want things to change but then they don’t want to keep it from them* (P1,FG3).

Embedded in the words “keep it from them” is an assumption that partners have a ‘right to know’. There was also concern as to when the best time to tell their partner would be:

*“You don’t want to say it too soon cos then people are more likely to leave sooner because*.

*they don’t like have that like strong connection but [laughter] the longer you wait the less likely is someone is to be like ah I don’t like you that much anyway”* (P3,FG2).

Interestingly, two of the FGs cited the film ‘Five Feet Apart’ as a source for their opinions, even though the film revolved around two teens with CF rather than CFSPID. Whilst one of the individuals acknowledged that the film was not the most accurate portrayal, they still used it as a basis for their opinions, showing the influence that popular media can have on this age group. A small proportion of participants also articulated worry about the effects of disclosing their CFSPID status to their partner, without being specific. However, one participant mentioned the potential *‘burden’* that a person with CFSPID might place on their partner (Participant 10, FG 2). The word *‘burden’* is emotive and implies a higher level of support and care is needed from the partner than they can cope with.

#### Disclosure in relationships: consideration for partner

Throughout discussions of when they would prefer to be told by their partner of their CFSPID diagnosis, there was a sense of empathy for their partner, and participants suggested it was ultimately their partner’s choice. However, as the receiver of the news, participants held a range of views about when they should be told:

*“I think obviously it’s up to them*,* but I think it should be done as soon as possible just so the other person is aware*,* like knows what they’re getting into”* (P10,FG3).

Whilst this was phrased as obvious that the decision lies with their partner with CFSPID, they still focused on when they would want to know. In this case, earlier rather than later, as the words “getting into” suggest a need for more consideration than a conventional relationship path again suggesting a sense of this relationship being different - “getting into” is usually part of a phrase suggesting a risk or a challenging situation. This idea was supported by others in the group.

Whilst others agreed that timing is down to the individual with CFSPID, there was a final cut-off point by which participants felt information should be disclosed, which was once they were in a more settled relationship:

*“I think until you’re at a point where it might actually affect your relationship*,* i.e. you’re planning long-term things or you want to have children or something. I think until then it is ultimately their decision whether they tell you”* (P4,FG4).

This suggestion that they would want to know only when it had a tangible effect on their future plans was common across FGs. This idea was also reflected by the same individual from the point of view of someone with CFSPID, who suggested that, using ‘*logic*’(P4,FG4), they would only tell their partner if they believed it was going to be a long-term relationship. Whilst only one individual stated that they had no specific time preference for disclosure, a relatively common idea across FGs was that it would depend on the individual or the relationship’s characteristics, such as openness of communication. One person pointed out that the receiver of the news might have different reactions depending on when they were told:

*“I think it depends how far along you are in the relationship*,* because if it’s like early days*,* you might like be more likely to end it or something. Where I feel like if you’re further down I would stick through with them more”* (P8,FG3).

This participant was the only person who expressed a view from the perspective of a partner of a person with CFSPID, regarding the timing and likely end of their relationship. However, the same opinion was expressed more commonly from the viewpoint of the person with CFSPID across all groups. It could perhaps be inferred from this that the person with CFSPID might be more worried that their partner would end the relationship than the actual risk of this happening. A number of participants expressed the view that if they were in a relationship, they would be worried if they believed their partner did not trust them enough to tell them something as important as a CFSPID. This implies that participants valued honesty in relationships, and this was echoed by one participant, who expressed their happiness if their partner with CFSPID trusted them enough to disclose their diagnosis.

#### Divergence from relationship norms: concerns about supporting partner

Throughout the discussions, there was an expectation that when in a relationship, you should be there for your partner. However, with the addition of a CFSPID diagnosis, there were questions across all FGs as to whether people felt able to fully do this. People believed that more support would be required than they would expect in a typical relationship, again suggesting a difference:

*“The level of support that they’d require from you would be obviously a lot more than if they didn’t have*,* so you would have to factor that into your decision making”* (P10, FG2).

Although participants had been told that the long-term consequences of CFSPID were unknown and that symptoms may not develop, they still assumed that supporting a partner with CFSPID would require more effort and they believed others would make this assumption too. It was unclear whether the need for extra support was a result of living with uncertainty or if there was an incorrect assumption that the individual with CFSPID would be symptomatic.

Furthermore, they believed that this may be an important factor that could affect relationship status:

*“I think I’d be concerned about how much responsibility that has*,* so if their condition did get worse very quickly*,* I’d think about how much time and how much - how it would affect me emotionally”* (P9,FG2).

Questioning how it might affect them suggests they would put themselves first, considering how it affected their life, rather than focusing on their partner. One individual even went so far as to say they would think ‘*selfishly*’ (P9,FG3). When asked to compare relationships to friendships, the view across FGs was that CFSPID would have a greater impact on a romantic relationship. Although there was a commonly held belief that they would want to support their partner if they had CFSPID, during two FGs, the belief emerged that they would require outside support themselves. Two participants suggested the dynamic of the relationship would change. One discussed having to fulfil more of a carer role if symptoms were present, and another suggested this could put a ‘*strain on the relationship*’ (P 3,FG4). However, whilst the majority did worry about the level of support their partner with CFSPID would require, there was one individual who expected, at some point, to care for their partner as part of the natural ageing process, and viewed this as equivalent:

*“If you’re like with them anyway*,* it shouldn’t really feel like a burden*,* cos with like growing old and stuff*,* you kind of expect one of you is going to have to look after the other or whatever*,* so like it’s not gonna be much different”* (P3,FG2).

####  Divergence from relationship norms: uncertain future

The uncertain future that accompanies a CFSPID diagnosis was a potential worry for the participants when considering romantic relationships. There seemed to be an inherent idea that for the relationship to be serious, some level of future planning was required, which would be hindered by the uncertainty of a CFSPID diagnosis:

*“It’ll be hard to make future plans like this*,* is what we’re gonna do in a year’s time or three years’ time*,* like the uncertainty that you don’t actually know what the situation is gonna be like in a few years’ time or whatever”* (P2,FG2).

Here, although they are staying with the idea of CFSPID having an uncertain prognosis, rather than viewing the person symptomatically, with a serious prognosis, CFSPID is still seen as different and not positive. This view regarding difficulties planning with a partner with CFSPID was shared by most participants across groups. A couple of participants went so far as to say that the uncertainty was worse than knowing something would definitely happen when questioned. There were also worries about the progression of CFSPID symptoms: *“There’s not like a guarantee – become a bit paranoid*,* like is that a sign? Is that a symptom?”*(P5,FG2).

This uncertainty may therefore lead to a person living in a constant state of anxiety, monitoring their partner with CFSPID for signs that might otherwise be seen as part of a normal life. There was also an assumption by a small number of participants across all FGs that a person with CFSPID would die sooner, interfering with the possibility of leading a normal life:

P2: *“At the same time*,* I think if you have plans like kids or anything*.

P10: *Yeah*.

P 2: *it wouldn’t be as possible if this person*,* your partner*,* dies early and then your kids don’t have a dad anymore*,* and it will be hard for these kids”* (FG 3).

However, only two participants considered the effect of the death on any children. Notably, the assumption of early death was made even though participants had only just been given information about CFSPID, which did not mention premature death as a symptom. The focus on the effects on their future children rather than just on themselves is explored in greater detail below.

Children seemed to be a major component of romantic relationships that came up spontaneously across all FGs. Whilst there was some confusion on the exact genetic mechanisms underlying CFSPID inheritance, there was a commonly held belief that, if possible, they would not want to perpetuate CFSPID:

*“I think you would be more hesitant in having kids with someone if it was something genetic because obviously you wouldn’t want it to carry on in your kids”* (P1,FG 3).

Although there is initial tentativeness here as to whether to have children, the latter half of the sentence suggests that there is certainty that they would not want their children to have CFSPID, so would potentially not procreate if this were a possibility. Further, this is presented as obvious, suggesting they assume it is a commonly held view. Most participants said they would want more information from their partner with CFSPID about the likelihood of passing it on to their children. In one FG, the idea of genetic testing in order to understand this likelihood was brought up, *‘I’d want to have the genetic testing … but like to know what the likelihood was’* (Participant 4, FG 4). Only a couple of participants in two of the FGs brought up the possibility of alternative ways to have children if people did not want to have biological children with their partner who had CFSPID:

*“I don’t think it stops them from having children altogether because they could discuss adoption and other ways of having children*”(P3,FG3).

As well as adoption, one participant brought up gene therapy as a potential way to reduce the chance of passing on the CF gene, whilst still being able to have biological children. There were still a couple of instances across the groups where confusion between CFSPID and CF occurred, as these terms were used interchangeably.

#### Impact of knowledge on relationship: changes in their attitudes towards a partner with CFSPID

Although participants realised they should not, or would not want to, view a partner with CFSPID differently, most participants believed it would happen to some extent:

*“I think there’s always going to be a subconscious*,* like it would change how I viewed the person knowing that”* (P5,FG3).

Although most participants did not suggest how their views would change, one individual suggested views would become more negative. However, two individuals in separate FGs believed their views would not change, as the person had not fundamentally changed:

*“Knowing about it doesn’t necessarily change who they are because they’ve grown up with this thing*,* it’s part of who they are*,* it’s influenced the way they’ve developed their personality and everything*,* and it couldn’t change the way I viewed them romantically because they’re the same person they’ve always been”* (P4,FG4).

Participant 4 had friends with health conditions (e.g. multiple sclerosis and coeliac disease).

As well as changing views, ideas regarding changes in the way people within the relationship would act, even unintentionally, were brought up:

*“Might see them as vulnerable even if you don’t want to*,* so you might treat them differently to someone who doesn’t have it”* (P8,FG4).

A partner’s vulnerability was cited as the cause of behaviour change in several other participants’ narratives. Individuals in two of the FGs said they would try to be *‘accommodating’* (P5,FG4) and would want to be more supportive without being *‘overbearing’* (P10,FG2), illustrating that although their behaviour changed, participants are keen that it’s not negative. There was some suggestion that the level of knowledge would affect reactions. An extreme view from one participant was *‘Not like a leper kind of thing but might be a bit more cautious”* (P1, FG2). Although this participant, who predominantly expressed negative views towards CFSPID, says “not like a leper”, this term is used in general communication to connote extreme social isolation and fear, so even using this as a comparison raises some concerns as lepers were separated from society, so even stepping back from this via the comment “not like a leper” suggests earlier stages of stigma could be experienced. However, across groups, it was believed that education could help with this by making people less *‘intimidated’* by the term CFSPID (P4,FG2). They would use their partner as one of the sources to seek out this information. Whilst lots of people believed that a CFSPID diagnosis would change their relationship, this was a very general view; there were few specific statements of what exactly would happen. For example, one individual mentioned not being able to go on *‘romantic dates’* without elaborating on what this meant for them (P7,FG2).

#### Impact of knowledge about CFSPID: reactions of people external to relationship

Only one of the FGs discussed how others might react to disclosure of a partner’s CFSPID status. Following a question specifically related to a friend’s reaction, someone said:

If they’re an actual friend they wouldn’t be negative about it all they could do is support you.

(P10,FG2)

This is interesting as by using the word ‘*actual*’ they seem to be differentiating the quality of the friendship. There seemed to be an assumption that a genuine, good friend would display loyalty and support their decision, perhaps regardless of their own thoughts, whilst they may expect a more negative reaction from someone they were less close to. Although this was a result of direct questioning, there was agreement within the FG on this and for family reactions:

*“I think they’d [family] be quite worried about me getting hurt like over the person was gonna get sick and die*,* that would be their objection to it”* (P2,FG2).

This comment associates families with loving relationships and a desire for family members to live stress-free and happy lives. The prospect of a family member’s partner being affected by CFSPID and the potential for that to cause the family member’s life to deviate from the expected ‘norm’ might also be the objection. This potential for distress was reflected in how some participants thought strangers might react:

*“just like*,* like why are you in a relationship with someone who’s sick*,* especially*,* if you’re like*,* it’s just kind of like– the idea of disease*,* not meaning in any specific context*,* makes me feel uncomfortable.”* (P2, FG2).

During the discussion, it was not clear whether this lack of understanding from strangers was based on the genetic label itself or the assumption that the individual with CFSPID could die sooner. Although different people, for example, family or friends, might question the relationship for different reasons, the overriding belief was that there is potentially some form of negative stigma surrounding a CFSPID diagnosis. However, at all times, discussions seemed to be less specific to CFSPID as people referred to disease in general, especially in reference to strangers’ beliefs.

####  Impact of knowledge about CFPSID on relationship: relationship avoidance

Although rare, some participants would not consider having a relationship with someone with a CFSPID diagnosis:

*“If you already had that kind of relationship with someone*,* then there’s a good chance it won’t matter by the time they actually tell you*,* but if you know before you start thinking that way*,* then you can like probably prevent you from like going there”* (P3,FG 2).

Even though there is no guarantee that a person with CFSPID will develop life-changing symptoms, the uncertainty associated with this label is enough to dissuade people from entering that relationship. This sentiment was echoed by another participant who believed that a.

stereotypical opinion might be: ‘Like why set yourself up for all that pain?’ (P2,FG2). This was related to age, with the individual believing that if someone was young, they would be less likely to date someone with CFSPID. Another participant in the FG voiced their agreement with this opinion. Although all this data comes from a single FG, it is concerning.

## Discussion

Our study is the first to explore how young adults will make sense of and react to the hypothetical disclosure of a novel, uncertain genetic result. Without previous knowledge about CFSPID, few participants held the line that, because they did not know anything about the condition, they should not make assumptions about it, echoing work conducted with YA exploring their knowledge of cystic fibrosis and sickle cell, where YA would report they knew nothing, but would still put forth responses based on tangential information confidently (Noke and Ulph 2014). This study echoes previous research suggesting that even when people think they have little knowledge about a topic, they are not passive or non-judgmental; they start drawing on any information and prior experiences to make sense of it. Participants in this study relied on existing knowledge of cystic fibrosis and illness models to make assumptions about the person with CFSPID, including fears around it being contagious. For many, this led to incorrect assumptions, which could be distressing for both the person with CFSPID and their peers. A similar pattern of sensemaking has been observed in children and YA trying to make sense of whole genome sequencing (Parfett et al. 2024), and it is present in the initial reactions of many parents when they are told that newborn screening has identified a need for further tests (Ulph and Bennett 2022). Interestly, our participants reflected that a general lack of understanding could make it harder for YAs with CFSPID to explain to their peers. This work develops this area by examining how YA respond to an uncertain diagnosis and found that although some discussions situated the prognosis as uncertain, participants would explicitly state that they found this state challenging and many would revert to a binary diagnostic model of sick or well similar to that shown by many parents adapting to CFSPID (Johnson et al. 2019; Tluczek et al. 2019) and young adults reacting to carriers (Bowen et al. 2025). There is a growing argument that attempts are made to alter predominant illness models to align them with what genetic advances are enabling health care systems to return in terms of results (Johnson et al. 2019) to help people understand and adapt to such information. This paper outlines how the harmful assumption that results from health care providers are normally linked to life-limiting conditions can trigger people to change their behaviour toward result recipients in stigmatising ways. This behaviour, which could trigger feelings of not belonging or of being different from your peer group, can be hugely detrimental and yet be avoided if we improve awareness of the types of results now generated and that we all have some unique DNA but much more in common.

Of note, although participants discussed CFSPID, there was little in their responses that was CFSPID-specific; the sense-making patterns and assumptions may well be transferable to other rare results from childhood screening/testing, which are not well known. Further research is urgently needed, given the rapid expansion in the testing for rare diseases, especially via newborn screening, to ensure that all the physical benefits of identification are realised whilst minimising any of the potential psychological harms (Ulph and Bennett 2022).

Furthermore, peers were keen to learn more and support the person with CFSPID, but struggled with knowing how appropriate it was to instigate discussions with the person with CFSPID – guidance here would be beneficial. Many talked about seeking further information via the internet; however, this has been shown to be problematic for parents of children with CFPSID (Johnson et al. 2019) and parents of carriers (Ulph et al. 2015). Although information about CFSPID is available on the CF trust website, there is no other information outside that provided by hospitals and academic papers.

Although the majority of responses portray a desire to be a good friend or partner, underpinning the narratives around their behaviour, the appropriateness of partners knowing, etc., is the sense that CFSPID would be seen as a difference and as such, they would be treated differently – the first part of Link & Phelan’s (2001) concept of Stigma. There was a sense that they would need more support, and relationship futures seem less certain. Indeed, the concept of a burden from different standpoints was a central thread through the data. Burden arose in terms of the burden of explaining CFSPID to peers and not wanting to burden them with support needs; friends not wanting to burden the YA with CFSPID with questions, assumptions that their friend would be carrying a burden and need support, etc. Before the study, they were only told that CFSPID was a newborn screening result where the impact and treatment needs were unclear. After giving their initial thoughts, they were provided with information about CFPSID, telling them: “This means [people with CFSPID] probably don’t need to be treated like they have CF at present, but may go on to develop symptoms of CF later in life. Healthcare professionals, however, cannot say at present when and what symptoms may develop in a child with CFSPID, if they develop at all”. However, despite this, the participants often portrayed people with CFPSID as currently symptomatic and would treat them like they have CF. Whether this is because they confused it with CF or because it was based on more general perceptions that being given a label after testing always suggests something serious is unclear; what was clear was that negative stereotypes were being used in this situation. It has already been argued that uncertain results provided via newborn screening clash with common cognitive models of health and healthcare (Johnson 2025). Specifically, none of the discussions mentioned the idea that the CFPSID designation is a positive scenario in which people can be followed up and monitored to ensure optimal health, which is the central goal of such designations. Rather than a criticism of our participants, we believe this suggests that, without public engagement to explain the meanings and benefits of these new types of screening designations, the benefits that clinicians see of returning these results will not be understood, as these are based on a preventative model of healthcare that people do not seem to be using when interpreting this information.

A tiny minority voiced that they would avoid romantic relationships. Placing it in context is important – it occurred in one group only, and the views came largely from one participant, however, this was a small scientifically literate sample discussing in a group setting with judgement from peers, and any such views are important to understand and attend to, given the negative impact they can have directly through behaviours towards the person, and indirectly by influencing other peers. Indeed, it is likely that peers will speak to other peers, and our data suggest that their views can be driven by peer responses, however incorrect. As stated above, our work fits with that of others, which suggests that in a scenario of discussing a poorly known condition, YAs will draw on any information, however tangential (Noke and Ulph 2014; Parfett et al. 2024). In this scenario, participants formulated a picture of someone with CFSPID as currently being symptomatic which led to behaviours such as distancing themselves from the person with CFPSID or treating them differently. Of note, as part of the ethical recruitment process, participants were given pre-study information about the topics of the FGs and were presented with information about CFPSID in the FGs, and yet still views based on tangential knowledge schemas were used. However, this could be because the participants were students who were participating for course credits and so may have paid less attention to pre-study information. Educational campaigns, which have been shown to reduce stigma previously (Couto et al. 2024), may still be effective if researched and designed appropriately. Careful design is necessary as research has shown that the results from newborn screening clash with conventional health knowledge models, which are symptom-diagnosis-prognosis driven, which adults can find challenging to process (Musa and Ulph 2026). As reported above, some participants would find it challenging when a genetic result had been given, but their peer “looked healthy”. We know this is the case for parents receiving newborn screening results for babies that look well, and it is also a challenge for those with unseen disabilities.

Echoing what is known in the literature about YA finding it stressful to disclose diagnoses to peers (Kaushansky et al., 2017), participants believed it would be hard for YA with CFSPID to disclose this information to peers due to a lack of awareness in the general public, discussions about how this results in fear, and concern about potential negative reactions driven by this lack of awareness. Unfortunately, the above suggests that this concern may be warranted. This concern has also been found directly in reports from parents of children who are carriers (Ulph et al. 2014), but also implicitly in the communication content and style of parents revealing diagnoses (Middleton et al. 2018).However, our participants also discussed beliefs around a friend or partner withholding CFSPID-related information from them, which they interpreted as negative behaviour.

### Strengths and limitations

The qualitative design is a strength, given arguments that it is better able to explore the impact of genomic labels (Biesecker et al., 2013). The current study employed an experienced CFSPID qualitative researcher who assisted with the development of the FG schedule. The use of prompts and exercises appeared to help participants contribute as it took focus away from the moderator and onto the group itself (Kitzinger 1995). A technical issue during FG 1 led to it not being audio recorded and, therefore, lost data which may have provided further insight and valuable information regarding our research. We, however, ran a fourth FG to compensate for the loss of data. Due to the Participant Information Sheet that had to provide a sufficient description of our research to comply with ethical approval, we did not include a small number of references where participants expressed knowledge which clearly originated from the PIS. This may be criticised; however, it ensured the analysis was better aligned to the study’s interest in people’s reactions and understanding of an unknown diagnosis, who in reality would not have received pre-disclosure information about CFSPID. We recognise that all research examining knowledge has the potential to be influenced by what is given pre-study, and so we wished to minimise this influence on our analysis. A limitation is the use of psychology students from one university, resulting in a more homogenous sample in respect of age, social class, and education level. Caution must also be taken as the participants are reacting to hypothetical situations. However, as argued previously, they still provide insight into the underlying beliefs and where people are drawing their knowledge from, indicating what is needed to reduce stigmatisation or distress (Bowen et al. 2025). Also, although the responses linked to interactions with peers and romantic partners were hypothetical, the sense-making work they undertook is not; we accept that sense-making related to a known person may differ.

### Future research

It is important that research is conducted into the experience of individuals with CFSPID across childhood to provide firsthand accounts of how this designation affects individuals and their families. Our participants held stigmatising beliefs due to misconceptions. Campaigns to increase awareness via education settings may improve peers’ awareness of CFSPID. Our research provides evidence that peers will seek out further information after an individual with CFSPID discloses their result. In conjunction with increasing awareness, accurate and trustworthy online information sources need to be provided for individuals with CFSPID and their peers. Future research involving individuals with CFSPID to discuss what they want to be available online for their peers is crucial. Exploring how other YA with similar results use the internet to support their disclosure to peers would also be appropriate. The population used in the sample, as previously stated, was suitable for exploring how young adults would respond to CFSPID. However, it would be appropriate to explore if the same patterns of understanding are found in non-university-educated YAs.

## Conclusion

There is little evidence about what impact the CFSPID label may have on individuals. This research aimed to identify how YAs attempted to make sense of this novel result and to predict the guidance needed to mitigate any adverse impact individuals with CFSPID may face. Our findings show that in a hypothetical situation, the reaction of peers and others is at best neutral but can be negative due to a reliance on ‘CF’ knowledge. If this is similar to real-life situations, this suggests individuals with CFSPID may be stigmatised. A priority for future work is to conduct research with individuals with CFSPID to understand their experiences of disclosure, information needs, and what they wish were available to support them in their peer disclosure.

## Supplementary Information

Below is the link to the electronic supplementary material.

Supplementary Material 1

Supplementary Material 2

## Data Availability

Data is available upon reasonable request.
