# Supplementary material for: Young adults’ understanding of, and reaction to, those with uncertain genetic results
Source: J Community Genet. 2026 Oct 20;17(6):127. doi: 10.1007/s12687-026-00957-w (PMC13683284; doi:10.1007/s12687-026-00957-w)
Supplement: Supplementary file 1 [file 12687_2026_957_MOESM1_ESM.docx]

**Exploring the attitudes young adults hold towards people with uncertain diagnosis outcomes and their willingness to engage with them**

**Participant Information Sheet (PIS)**

You are being invited to take part in a research study that will explore young adults’ views on how receiving a new type of uncertain diagnosis may impact on peer and romantic relationships. This study is being conducted for an Undergraduate BSc psychology dissertation and will be undertaken by student researchers. Before you decide whether to take part, it is important for you to understand why the research is being conducted and what it will involve. Please take time to read the following information carefully before deciding whether to take part and discuss it with others if you wish. Please ask if there is anything that is not clear or if you would like more information. Thank you for taking the time to read this.

**About the research**

- **Who will conduct the research?**

Student researchers in the Faculty of Biology Medicine and Health, School of Health Sciences, The University of Manchester:

Kate Fifield, Eleanor Horowitz and Grace Christie.

Supervisor: Fiona Ulph, Faculty of Biology Medicine and Health, School of Health Sciences, The University of Manchester.

- **What is the purpose of the research?**

Newborn screening checks whether babies have one of nine rare conditions so that treatment can be started early. Although this has benefits for children with the conditions, a number of other results are returned to parents where the impact of giving the result is less clear. A new result which has started being given to parents is Cystic Fibrosis Screen Positive, Inconclusive Diagnosis (CFSPID). This is still a relatively unknown genetic disorder and healthcare professionals are unsure of the treatment for these children following this diagnosis. Preliminary research suggests that this diagnosis may have a psychological impact on some parents. What is unknown is how this diagnosis will impact on the children being given this label. One of the key drivers of this will be how their peers interact with them. As such we want to explore how young adults would respond to the scenario of a friend disclosing this diagnosis to them.

Within this we will be asking your opinions on hypothetical situations such as how you would seek information about CFSPID following a peer disclosure and the impact on peer and romantic relationships. You are being invited to participate as you are a young adult aged 18-25 at the University of Manchester where this project is being conducted. We are seeking to recruit approximately 36 young adults in total.

- **Will the outcomes of the research be published?**

This research will anticipate obtaining a greater understanding of what the public (young adults) may think about CFSPID and its impact on their peers. This study will take into consideration: the benefits, barriers and ethical issues that could arise for the population carrying the CFSPID diagnosis. Findings will be published in the students’ thesis and hopefully academic journals. We will not contact you with a summary of findings, but will happily provide this should you contact us.

- **Who has reviewed the research project?**

The project has been reviewed by the Division of Psychology and Mental Health Ethics Committee.

**What would my involvement be?**

- **What would I be asked to do if I took part?**

If you agree to take part, you will be invited to a focus group with approximately 12 other University of Manchester students. This will take place in Coupland building 1.

The focus group will take approximately 1 hour. It will be run by 2 of the students listed above.

When you arrive at the focus group we will check you have read the participant information sheet and give you an opportunity to ask any questions. If you are happy to continue, we will ask you to sign a consent form. Before the focus group starts, you will be asked to fill out a confidential anonymised questionnaire to enable us to describing the sample in our report.

The group will involve the researcher asking you to discuss topics including your views on CFSPID and its impact on peer relationships. During the group you will be provided with information about CFSPID to explore your views further. **You are not expected to have any prior knowledge of this condition, as in the real world it is unlikely people will have heard of it. As such please do not try and educate yourself before the group.**

An audio device will record the focus group and this recording will be typed up into a transcript – the researcher will not type any information which may identify you. Once the transcript is checked the recording will be erased to maintain the participant’s anonymity.

We do not envisage there being any risks, but you should know that it is not always possible to predict what other participants choose to discuss in focus groups. It is unlikely you will have had any experience of the topics we ask you to discuss – a diagnosis of CFSPID however, it is likely you will have had experiences as a peer, friend, in romantic relationships. As such there is a small risk that another participant could say something you find distressing. Support will be signposted to you if this is the case. Although the researcher will handle the data in a confidential manner, we cannot guarantee that other participants in the group will not disclose what is discussed. As such we urge participants to consider what they disclose to the group.

- **Will I be compensated for taking part?**

Upon completion of the study the researcher will award your 5 credits through the SONA system if you are an UG Psychology student. Otherwise there is no payment as such for participation.

- **What happens if I do not want to take part or if I change my mind?**

It is up to you to decide whether or not to take part. If you do not want to take part, do not sign up for a focus group. If you decide to take part you are still free to withdraw up to the point when your data is anonymised into the transcript. It will not be possible to remove your data from the project once it has been anonymised as we will not be able to identify your specific data. This does not affect your data protection rights.

Due to the group nature of a focus group, it is not possible to participate without being recorded. If you are uncomfortable with being recorded at any time, you are free to stop being recorded by leaving the room. The co-facilitator will come and check if you are ok.

**Data Protection and Confidentiality**

- **What information will you collect about me?**

In order to participate in this research project we will need to collect information that could identify you, called “personal identifiable information”. Specifically we will need to collect:

- An email address which will be used and stored in a secure database to set up the focus group. After participation, or if you do not want to take part, this will be destroyed.
- Your age so that we can describe the sample.

The recordings will consist of voice only and other individuals during the focus group

- **Under what legal basis are you collecting this information?**

We are collecting and storing this personal identifiable information in accordance with data protection law which protect your rights. These state that we must have a legal basis (specific reason) for collecting your data. For this study, the specific reason is that it is “a public interest task” and “a process necessary for research purposes”.

- **What are my rights in relation to the information you will collect about me?**

You have a number of rights under data protection law regarding your personal information. For example you can request a copy of the information we hold about you, including audio recordings.

If you would like to know more about your different rights or the way we use your personal information to ensure we follow the law, please consult our [Privacy Notice for Research](http://documents.manchester.ac.uk/display.aspx?DocID=37095).

- **Will my participation in the study be confidential and my personal identifiable information be protected?**

In accordance with data protection law, The University of Manchester is the Data Controller for this project. This means that we are responsible for making sure your personal information is kept secure, confidential and used only in the way you have been told it will be used. All researchers are trained with this in mind, and your data will be looked after in the following way:

Only the study team at The University of Manchester will have access to your personal information, but they will anonymise it as soon as possible. Your name and any other identifying information will be removed and replaced with an ID number. At the focus group we will assign you an ID number which will be on the questionnaire we ask you to fill out. When we transcribe the data we will use this rather than your name. We will anonymise the data fully by removing all names and references to location, etc. which could identify you. Your consent form and questionnaire will be retained for 5 years in accordance with the University regulations in separate locked filing cabinets in a locked office on campus. The group transcript will be transferred from an encrypted recorder onto a University of Manchester server. The recording will be destroyed once the transcript has been checked. The anonymised transcript will be kept on the same server.

If, during the study, we have concerns about your safety or the safety of others, we will inform the project supervisor who may either meet with you or will contact the relevant programme administrators in order to gain details to access support for you.

If, during the study, you disclose information about misconduct/poor practice, we have a professional obligation to report this and will therefore need to inform your course directors.

If, during the study, you disclose information about any current or future illegal activities, we have a legal obligation to report this and will therefore need to inform the relevant authorities.

Individuals from the University, the site where the research is taking place and regulatory authorities may need to review the study information for auditing and monitoring purposes or in the event of an incident.

**Regarding the audio-recordings:**

- The recordings will be transcribed by a member of the research team.
- Personal identifiable information will be removed in the final transcript
- The recordings will be destroyed after the transcript has been checked.
- Members of the research team will have access to the recordings.

Please also note that individuals from The University of Manchester or regulatory authorities may need to look at the data collected for this study to make sure the project is being carried out as planned. This may involve looking at identifiable data. All individuals involved in auditing and monitoring the study will have a strict duty of confidentiality to you as a research participant.

**What if I have a complaint?**

- **Contact details for complaints**

If you have a complaint that you wish to direct to members of the research team, please contact:

**FIONA ULPH:** [**Fiona.ulph@manchester.ac.uk**](mailto:Fiona.ulph@manchester.ac.uk) **0161 271979**

**If you wish to make a formal complaint to someone independent of the research team or if you are not satisfied with the response you have gained from the researchers in the first instance then please contact:**

The Research Governance and Integrity Officer, Research Office, Christie Building, The University of Manchester, Oxford Road, Manchester, M13 9PL, by emailing: [research.complaints@manchester.ac.uk](mailto:research.complaints@manchester.ac.uk)  or by telephoning 0161 275 2674.

If you wish to contact us about your data protection rights, please email [dataprotection@manchester.ac.uk](mailto:dataprotection@manchester.ac.uk) or write to The Information Governance Office, Christie Building, The University of Manchester, Oxford Road, M13 9PL at the University and we will guide you through the process of exercising your rights.

You also have a right to complain to the [Information Commissioner’s Office](https://ico.org.uk/concerns) about complaints

**Contact Details**

If you have any queries about the study or if you are interested in taking part then please contact the researcher(s):

Kate Fifield**:** [kate.fifield@student.manchester.ac.uk](mailto:kate.fifield@student.manchester.ac.uk)

Grace Christie: [grace.christie@student.manchester.ac.uk](mailto:grace.christie@student.manchester.ac.uk)

Eleonor Horowitz: [eleanor.horowitz@student.manchester.ac.uk](mailto:eleanor.horowitz@student.manchester.ac.uk)

**FIONA ULPH** [**Fiona.ulph@manchester.ac.uk**](mailto:Fiona.ulph@manchester.ac.uk) **0161 271979**
